# Living in Your Skin: Microbes, Molecules, and Mechanisms

**DOI:** 10.1128/IAI.00695-20

**Published:** 2021-03-17

**Authors:** Mary Hannah Swaney, Lindsay R. Kalan

**Affiliations:** a Department of Medical Microbiology and Immunology, School of Medicine and Public Health, University of Wisconsin, Madison, Wisconsin, USA; b Department of Medicine, School of Medicine and Public Health, University of Wisconsin, Madison, Wisconsin, USA; University of Pittsburgh

**Keywords:** host-microbe interaction, microbial ecology, skin microbiome, specialized metabolite, wound infection

## Abstract

Human skin functions as a physical, chemical, and immune barrier against the external environment while also providing a protective niche for its resident microbiota, known as the skin microbiome. Cooperation between the microbiota, host skin cells, and the immune system is responsible for maintenance of skin health, and a disruption to this delicate balance, such as by pathogen invasion or a breach in the skin barrier, may lead to impaired skin function. In this minireview, we describe the role of the microbiome in microbe, host, and immune interactions under distinct skin states, including homeostasis, tissue repair, and wound infection. Furthermore, we highlight the growing number of diverse microbial metabolites and products that have been identified to mediate these interactions, particularly those involved in host-microbe communication and defensive symbiosis. We also address the contextual pathogenicity exhibited by many skin commensals and provide insight into future directions in the skin microbiome field.

## Abstract (precis)

Human skin functions as a physical, chemical, and immune barrier against the external environment while also providing a protective niche for its resident microbiota, known as the skin microbiome. Cooperation between the microbiota, host skin cells, and the immune system is responsible for maintenance of skin health, and a disruption to this delicate balance, such as by pathogen invasion or a breach in the skin barrier, may lead to impaired skin function.

## INTRODUCTION

The debate over whether bacteria residing on the skin cause disease or simply are mere bystanders has persisted for well over a century (1, 2). Before the germ theory of disease was accepted and bacteria were successfully cultured from human tissues, Semmelweis dramatically reduced the mortality rate of pregnant women by simply introducing hand washing in his clinic (3), and in the late 1800s, Lister pioneered antiseptic surgical procedures (4). By the early 1900s, the idea that humans are colonized by microorganisms in the hours after birth was at the fore. However, given the inability to fulfill Koch’s postulate in some dermatological diseases, the significance of the skin microbiota in health and disease remains an open question.

The skin is a multifunctional organ. As our primary environmental barrier, it protects us from mechanical impacts, prevents dehydration but also cools us off when we are hot, and allows us to perceive the world around us through a dense interface of different nerve endings. The skin is absolutely critical to our survival. Arguably, one of the most important functions of the skin is to protect against infection by blocking invading pathogens. This is accomplished through the organization of three specialized layers, the hypodermis, dermis, and epidermis. The epidermis forms the outermost waterproof layer consisting of the stratum corneum (SC), stratum granulosum (SG), stratum spinosum (SS), and stratum basale (SB) (5, 6). Through a process known as cornification, keratinocytes differentiate as they migrate through the strata, transforming into flat, anucleated corneocytes. Forming the acidic cornified envelope (CE), corneocytes contain tightly packed keratin filaments embedded within lipid layers and are tightly joined together by corneodesmosomes. The process is completed when cells are eventually shed as a result of desquamation (5, 7). In addition to the physical barrier created, skin cells selectively respond to the presence of skin pathogens to induce production of antimicrobial peptides (8, 9).

**FIG 1:**
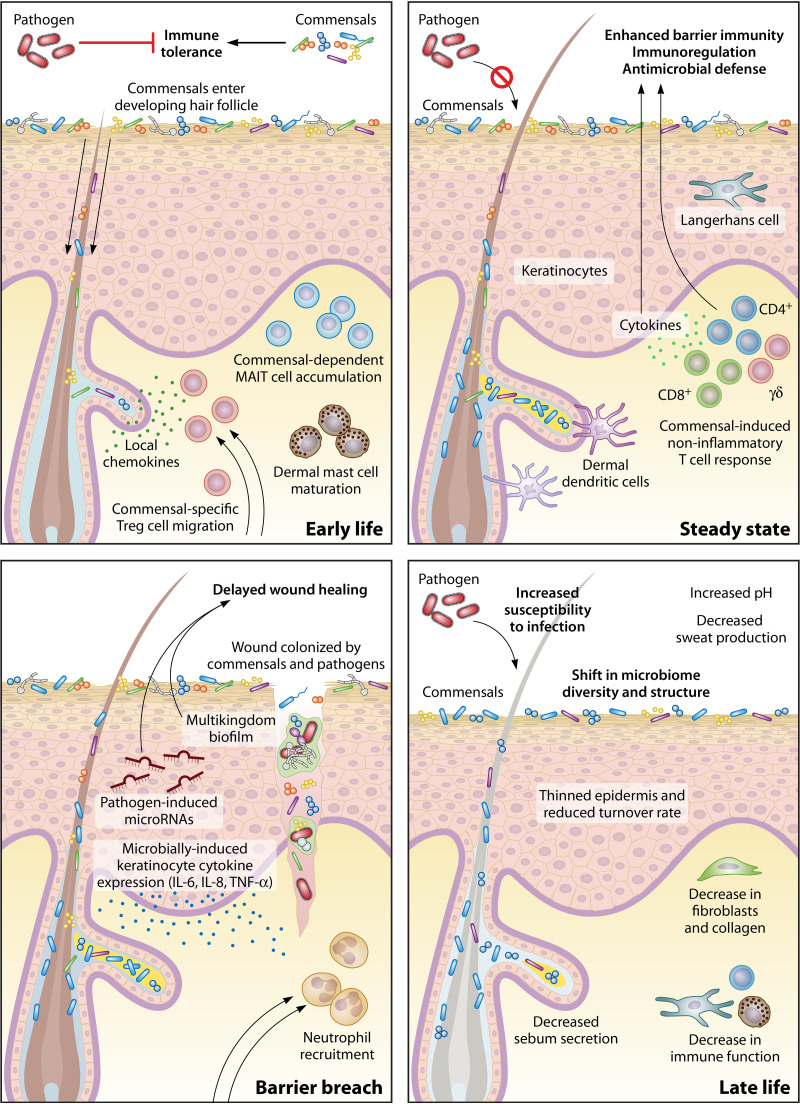
Skin state and age influence host-microbiota interactions. In early life, colonization of neonate skin by commensals contributes to development of the immune system and commensal-specific tolerance. During steady state, the skin microbiota is sensed by skin cells and immune cells, inducing a noninflammatory T cell response that leads to enhanced barrier function. After a skin barrier breach, a distinct wound-healing cascade is initiated. However, colonization of the wound bed and biofilm formation by commensals and occasionally pathogens can disrupt the highly coordinated wound-healing response and lead to delayed healing. Lastly, as the skin ages in late life, a gradual loss of function in the physical, chemical, and immune properties of the skin leads to a shifted skin microbiome and susceptibility to infection.

Although the skin functions to keep harmful microbes out, it is also estimated to provide nearly 30 m^2^ of diverse microbial habitat (10). Across the moist, sebaceous, or dry microenvironments of the skin are distinct microbial communities encompassing bacteria, fungi, viruses, and microeukaryotes. In this review, members of these communities are referred to as skin commensals, which we define as microorganisms whose interactions on the skin are either neutral or beneficial. Collectively, the skin microbiota have been implicated in the healthy development of an intact barrier throughout the life span. For example, cooperation between commensal microbes and hair follicles during a defined window of development in early life is required for skin immune cell homeostasis and mediating tolerance to commensal bacteria (11, 12). In aging individuals, physiological changes to the skin are concomitant with changes to the microbiome and a loss of phylogenetic diversity (13) (Fig. 1). That the skin represents a changing ecosystem intimately tied to its microbiome means that skin microbiome signatures are the most accurate predictor of chronological age, superior to oral or gut microbiome signatures (13–16). Members of the skin microbiome are not merely hitchhikers either, but active participants helping to maintain the integrity of the skin barrier. Modulation of the cutaneous inflammatory response, promotion of epidermal differentiation, and enhancement of wound healing are only a few examples of critical barrier function processes that the skin microbiota have been implicated in (11, 12, 17, 18).

A hallmark trait of many skin diseases is an aberrant microbiome accompanying a breakdown in skin barrier function and increased risk of infection. Occupation of skin niches by the microbiome provides protection against invading pathogens by both direct and indirect mechanisms, such as by competitive exclusion (indirect) and by the biosynthesis of microbiome-derived antimicrobial molecules (direct) (19, 20). However, the role of commensals residing on the skin is context dependent, with some species pivoting between healthy and diseased states. This reservoir of potential infectious agents is kept in a delicate balance by forces stabilizing the microbiome community itself and interactions with resident skin cells and host immunity. Yet, dermatological disease affects at least 25% of the population in the United States, with skin and wound infection accounting for the largest disease burden (21). Chronic wound infection is a growing problem, some estimating health care costs of a staggering $95 billion per year (22). A hallmark feature of these wounds is the presence of skin commensals, embedded within new communities and a vastly different environment.

In this review, we discuss the role of the skin microbiome, focusing on bacteria and fungi in a context-dependent manner. We explore the spectrum of microbe-host and microbe-microbe interactions that influence skin health and their influence on barrier function, including modulation of wound-healing pathways. Chemically diverse microbiome-derived metabolites are emphasized as critical mediators within skin microbial communities, and we highlight the ways in which members of the skin microbiota engage in defensive symbiosis.

## COMMENSAL VERSUS PATHOGEN RECOGNITION ON THE SKIN

Knowledge of skin immune function is largely based on the inflammatory immune response to pathogens. However, the majority of microbe-immune interactions on healthy uncompromised skin involve the resident skin microbiota, which have evolved intimately with the immune system to maintain a symbiotic host-microbe relationship. Thus, the microbiota, skin cells, and the innate and adaptive immune system must work in concert to establish and maintain the skin barrier. Importantly, the immune system must distinguish the resident skin microbiota from pathogenic microorganisms without eliciting an inflammatory response, ultimately promoting commensal tolerance.

### Immune system education.

Critical to proper immune function and commensal tolerance is the development of the immune system. Studies have shown that during a defined window of neonate development, regulatory T cells (Tregs), which are necessary for the establishment and maintenance of immune homeostasis (23), accumulate in the skin and establish immune tolerance to skin microbiota (11, 12). Colonization of neonatal but not adult mice with the skin commensal Staphylococcus epidermidis resulted in enriched commensal-specific Tregs and attenuated inflammation upon subsequent challenge with S. epidermidis, underscoring the importance of colonization timing in establishing immune tolerance (12). This tolerance, facilitated by the wave of Tregs into neonatal skin, was dependent on the coordination of neonate hair follicle development, commensal colonization, and local chemokine production (11) (Fig. 1). However, tolerance is not extended to just any bacterial species encountered during neonate development. The host immune system has been shown to discriminate between commensal (S. epidermidis) and pathogen (Staphylococcus aureus) through interleukin-1 (IL-1) signaling induced by S. aureus alpha-toxin, which in turn limits pathogen-specific Tregs (24). Thus, the developing neonatal immune system selectively promotes commensal tolerance while preventing tolerance to a harmful skin pathogen.

### Immune system homeostasis.

Homeostatic immunity, defined as the adaptive host responses to commensal microbiota that are developed and established in the absence of inflammation (25), plays an essential role in commensal tolerance on the skin. As an example of homeostatic immunity, colonization by the commensal S. epidermidis in mice has been demonstrated to induce effector T (Teff) cells through the coordinated response of commensal-sensing skin-resident dendritic cells. The robust accumulation of Teff cells occurs across various skin sites and is importantly uncoupled from inflammation, leading to enhanced barrier immunity (26). Further, an additional defined interaction exemplifying homeostatic immunity involves nonclassical major histocompatibility complex (MHC) presentation of commensal-derived antigen. In mice, presentation of S. epidermidis peptides by nonclassical MHC molecules to Teff cells results in a systemic noninflammatory and pleiotropic immune response, distinct from a pathogen-induced response. This response is characterized by protective immunity, immunoregulation, and tissue repair (18). At the same time, S. epidermidis has been shown to play a critical role in promoting IL-1 signaling and subsequent Teff cell function in murine skin, and this control is tuned according to the local inflammatory milieu (27). Further, the cutaneous immune response against pathogen S. aureus can be balanced and directed by the presence of S. epidermidis (28, 29). Expression of MHCII by keratinocytes has also been shown to mediate homeostatic immunity to commensal colonization, specifically through the control of commensal-induced Th1 cells (30). Overall, distinct yet intertwined commensal-mediated mechanisms are responsible for calibrating the immune system to respond to fluctuating commensal signals and invading pathogens (Fig. 1).

In addition to colonization by bacteria under steady-state conditions (26, 27), cutaneous colonization by commensal fungi has been demonstrated to induce noninflammatory skin immune responses, with fungi inducing a highly polarized and Th17-dependent response. However, in an experimental psoriasis model, recall responses to these fungal commensals following preexposure actually promoted skin inflammation (31), highlighting the importance of studying skin barrier immunity and pathologies in the context of commensal microbiota exposure.

## METABOLITE-MEDIATED MICROBIOME-HOST INTERACTIONS

The majority of microbial metabolites produced by the skin microbiota, and consequently their functions, are unknown. While the skin is generally perceived as nutrient poor, resident skin microbes have evolved to utilize the limited nutrients available on the skin, which include proteins, lipids, and other host-derived molecules, to support their growth and supplement microbial metabolism (32). As such, the microbiota produces a range of molecules that may be synthesized *de novo* or metabolized from host-derived molecules, providing the opportunity for microbe-host cross talk. These interactions between commensal microbiota and the host can be bi-directional, with microbial metabolites mediating skin and immune cell function or with host metabolites affecting microbial metabolism, both of which have consequences for maintenance of the microbiome and skin health. Because the skin microbiome has been found to extend to subepidermal compartments of the skin, including hair follicles, the dermis, and adipose tissue (33), metabolite-mediated interactions occur not only at the skin surface but deep within skin layers, as well.

### Metabolite-mediated interactions with the immune system.

A dominant referee of host-microbe interactions is the immune system, which must monitor and continually sample the multitude of both host- and microbe-derived molecules within the skin environment. An example of this necessary immune surveillance is the sensing of metabolites by MAIT cells. Distinct from traditional T cells that recognize peptidic antigen presented by MHC, MAIT cells are restricted by the MHC class I-related MR1 molecule, which captures and presents small metabolites. In particular, MAIT cells are characterized by their recognition of transient intermediates of riboflavin (vitamin B_2_) biosynthesis (34), which is broadly conserved among bacteria and fungi but not mammals (35).

Interestingly, MAIT cells are absent in germ-free mice (17, 36), indicating the requirement for microbial colonization in the expansion of MAIT cells in the periphery tissue. While numerous studies have demonstrated that MAIT cells are protective against pathogens (37, 38), they have also been shown to be activated by extracellular metabolites from commensal microbiota (17, 39, 40). Recent work has demonstrated that in mice, skin association of S. epidermidis or 5-OP-RU (transient riboflavin biosynthesis intermediate) results in skin-resident MAIT cell expansion in an IL-1- and IL-18-dependent manner (17). However, association with an S. epidermidis riboflavin biosynthesis mutant is unable to induce the same response. On the skin, the majority of species within the dominant bacterial skin taxa *Corynebacteriaceae*, *Propionibacteriaceae*, and *Staphylococcaceae* encode riboflavin biosynthesis; therefore, MAIT cell-specific immunity through recognition of riboflavin biosynthesis intermediates is likely to play an important role in skin barrier heath.

While the majority of studies have focused on 5-OP-RU as the predominant MAIT cell activator, MR1 is capable of binding other small cyclic molecules, distinct from those derived from riboflavin biosynthesis. This growing list includes bacterial metabolites (41, 42), drug and drug-like molecules (43), and vitamin B_9_ (folate) derivatives (34), which can be either activating or inhibitory for MAIT cells. Further studies are needed to determine the identity of other microbial metabolites that bind MR1 and their functional role in MAIT cell-mediated immune homeostasis in the skin.

### Metabolite-mediated interactions with host skin cells.

Keratinocytes, the cells that form the outermost layer of the skin, exist in close association with the skin microbiota and are thus poised to sense alterations in the skin barrier and its microbial communities. Failure to do so could result in microbial overgrowth and an unchecked immune response, disrupting the fine-tuned balance of this host-microbiota relationship.

**FIG 2:**
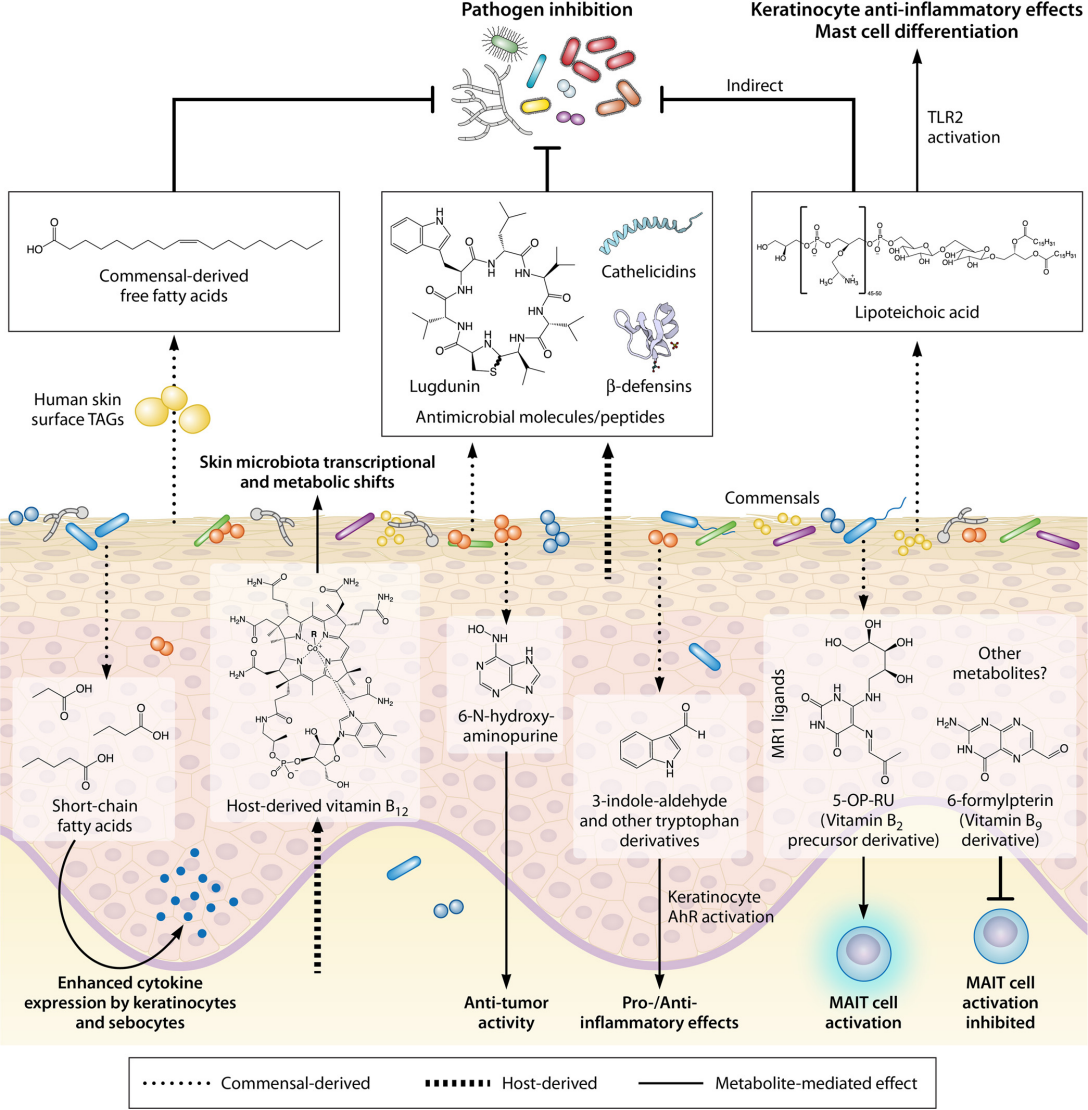
Metabolite-mediated interactions in the skin. The skin microbiota, which colonizes the skin and its appendages both epidermally and subepidermally, produces chemically diverse metabolites, which may be synthesized *de novo* or metabolized from host-derived molecules. These metabolites confer numerous benefits for the host, including pathogen inhibition, immune education and homeostasis, or even anti-tumor activity. However, under certain conditions, microbial products may promote an inflammatory response. Further, metabolites derived from the host can modulate commensal metabolism, demonstrating the bi-directionality of host-microbiota interactions on the skin.

Toll-like receptors (TLRs), which are expressed by keratinocytes and other skin cells such as fibroblasts and adipocytes, are involved in skin immunosurveillance and function to recognize microbial components, subsequently instructing innate and adaptive immune responses (44). The skin commensal Cutibacterium acnes has been demonstrated to produce short-chain fatty acids (SCFA) when grown under hypoxic conditions and in the presence of exogenous lipids. These SCFAs inhibit the activity of keratinocyte histone deacetylase, an enzyme involved in epigenetic control, leading to enhanced responsiveness to TLR activation and cytokine expression (45). Through a similar mechanism in the lipid-producing sebocytes of the sebaceous gland, *C. acnes* SCFA amplify TLR activation responsiveness and also directly activate free fatty acid receptors, both of which lead to enhanced proinflammatory cytokine expression (46). These results support a model where *C. acnes* overgrowth in the lipid-rich, anoxic hair follicle leads to increased inflammation, as is seen in acne pathogenesis, and importantly, shows how skin microenvironmental conditions may affect microbial metabolism and drive loss of immune tolerance to commensals. In addition, S. epidermidis lipoteichoic acid (LTA), a Gram-positive bacterial cell wall component, is a known ligand of TLR2 (47). Through TLR2-dependent mechanisms, LTA has been identified to have an anti-inflammatory effect on keratinocytes (48) and to stimulate production of keratinocyte stem cell factor (SCF) (49). It is also critical for mast cell differentiation (49) and to protect against viral vaccinia skin infection (50). These studies reinforce the multifaceted role of the microbiota and its metabolic products as mediators of cutaneous immunity (Fig. 2).

Dead keratinocytes and broken keratin in the stratum corneum provide a source of amino acids on the skin surface, which may then be metabolized by the microbiota and recognized by host cells. In a mouse model of atopic dermatitis (AD), the microbiota-derived tryptophan metabolite indole-3-aldehyde was shown to alleviate inflammation through binding and activation of the keratinocyte aryl hydrocarbon receptor (AhR) (51) (Fig. 2). This subsequently suppressed expression of thymic stromal lymphopoietin (TSLP), a cytokine that can contribute to aberrant immune responses in AD. Similarly, many species of the common fungal commensal *Malassezia* were identified to produce indolic metabolites that exhibited high AhR-activating capacity (52). However, these bioactive molecules were associated with pathogenic potential of the *Malassezia* strains in the context of the *Malassezia*-associated skin diseases seborrheic dermatitis and pityriasis versicolor, underscoring the intricate network of interactions that dictates the balance between skin homeostasis and disease.

While the majority of microbial metabolites are most likely produced as a result of evolutionary adaptation to the host skin niche, there is evidence of off-target host effects by products of microbial metabolism. For example, S. epidermidis produces the nucleobase analog 6-*N*-hydroxyaminopurine (6-HAP), which can inhibit DNA synthesis. Intriguingly, 6-HAP *in vitro* has selective antiproliferative action against tumor cell lines but not keratinocytes (53) (Fig. 2). Further, *in vivo* suppression of tumor growth induced by UV exposure can be conferred through skin colonization of an S. epidermidis strain that produces 6-HAP but not by a non-6-HAP-producing strain, demonstrating the potential for microbial metabolites to confer anti-tumor activity.

### Host-microbe cross talk.

While many of the characterized host-microbiota interactions describe the implications of microbial metabolites on host function, the effects of host-derived molecules on microbial metabolism are less well understood. Microbes will often preferentially uptake required nutrients if they are available in the environment. Kang et al. demonstrated that oral supplementation of healthy humans with vitamin B_12_, an essential cofactor in humans, resulted in a transcriptional and metabolic shift in the skin commensal *C. acnes* (Fig. 2). This shift was characterized by downregulation of genes involved in *C. acnes* vitamin B_12_ biosynthesis, as well as differential expression of genes with both known and unknown association to vitamin B_12_ metabolism (54). Host vitamin B_12_ supplementation also promoted the production of porphyrins by *C. acnes*, which is implicated in acne pathogenesis (55, 56). This demonstrates that host-acquired micronutrients are accessible to cutaneous microbes and that back-and-forth cross talk between the microbiota and host may play important roles in disease development. Further, this study highlights a potential role for vitamin B_12_ within skin microbial communities, especially considering that many bacteria require the cofactor but are unable to produce it themselves. We hypothesize that this is an area of importance for studying community dynamics in the skin microbiome.

## CONTEXTUAL HOST-MICROBIOTA INTERACTIONS

Under steady-state conditions, the immune system and skin commensals stably interact to promote healthy immune function. But what are the consequences when this delicate balance is disrupted, and what triggers this disruption? A recent study by Belkaid and colleagues provides insight, showing that under homeostatic conditions in mice, mycolic acid, which is present in the cell wall of almost all species of the *Corynebacterium* genus, elicits a distinct immune response characterized by IL-23 signaling and the accumulation of γδ T cells (Fig. 1). Similar to other skin commensals, this response is uncoupled from inflammation. However, under an inflammatory state or high fat diet, the immune response to the commensal Corynebacterium accolens was significantly altered, resulting in increased skin thickness and inflammatory cellular infiltrate, as well as a broad change in gene expression (57). Thus, control of skin immunity by commensal species is likely to be contextual and dependent on the inflammatory and metabolic state of the host.

**FIG 3:**
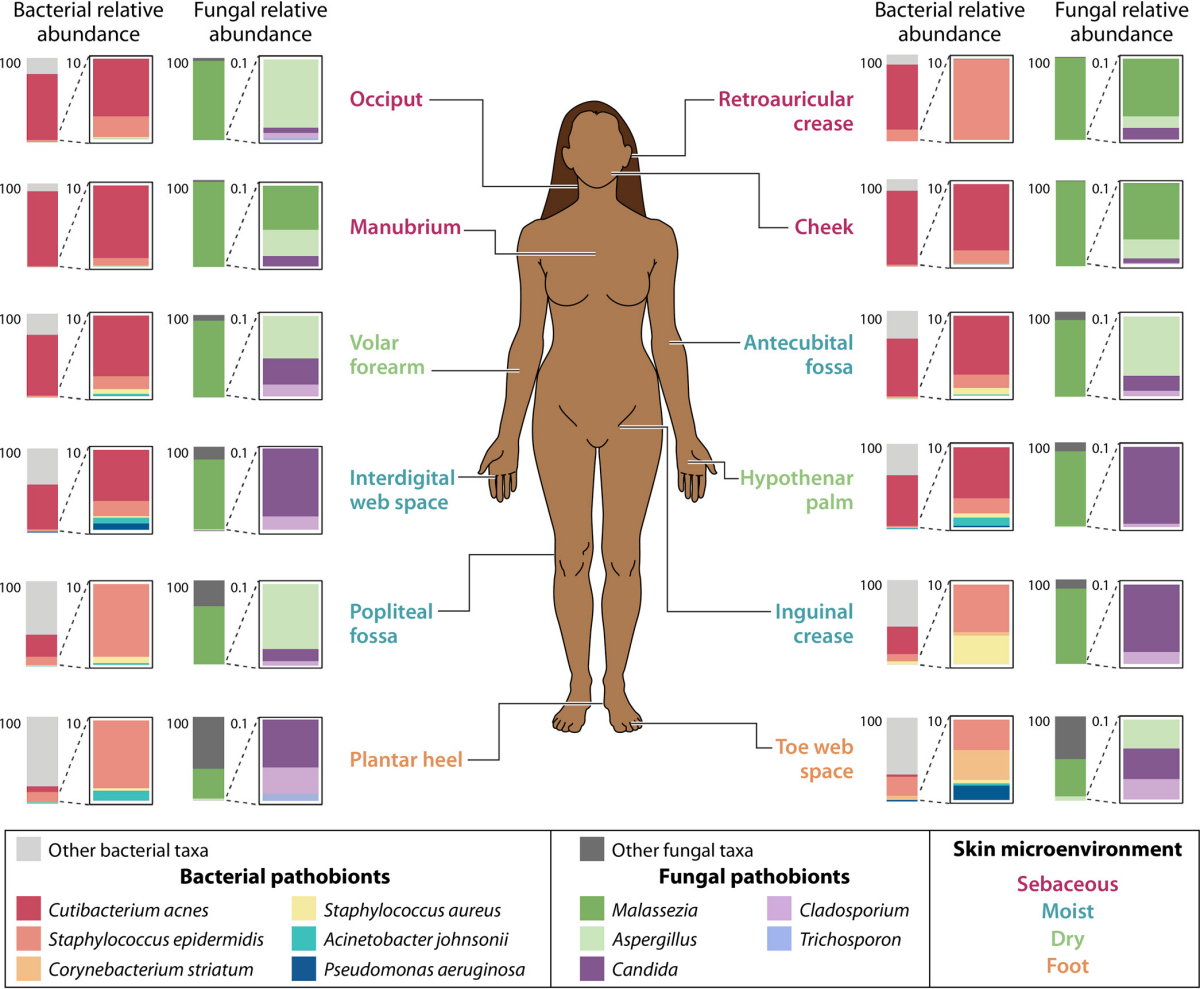
Pathobionts in the healthy skin microbiome. The average abundance of select pathobionts across 12 skin sites is shown. Sites are classified into sebaceous, moist, dry, or foot microenvironments. The data represent 11 to 17 healthy volunteer samples per skin site for bacterial abundance calculations and 2 to 9 healthy volunteer samples per skin site for fungal abundance calculations. Relative abundance data is from Tables S3 and S5 from reference 70.

Pathobionts are traditionally considered to be commensal microorganisms that can promote disease under particular host or environmental conditions. However, as suggested previously (58, 59), the term pathobiont insufficiently captures the broad and context-dependent effects that a particular microorganism may have on its host. Rather, microbial species often exist within a continuum between mutualism and pathogenicity, which can shift depending on factors such as host metabolic state, immunocompetence, or the presence of certain microbial partners. For example, S. aureus, a virulent pathogen that is a leading cause of community and nosocomial-acquired infections, asymptomatically colonizes 20 to 40% of the general population (60), demonstrating its range of potential pathogenicity (Fig. 3). Recent work suggests that cocolonization with other common commensals, such as *Corynebacterium* spp., may diminish expression of the S. aureus *agr* quorum sensing locus, which is key to production of numerous virulence factors (61). In doing so, this allows a shift to a state of commensalism, which could partially explain the persistence of S. aureus in the normal human microbiota. Conversely, coproporphyrin III production by *Cutibacterium* spp. has shown to induce aggregation of S. aureus and biofilm formation (62), which can allow survival of the bacteria under external stressors such as antibiotic exposure, desiccation, and immune attack (63). Thus, in the context of mutualism and pathogenicity, microbe-microbe interactions may have an unappreciated role. The mechanisms through which potentially pathogenic skin microorganisms respond to contrasting signals from the surrounding microbiota remains to be determined.

The isolation of skin commensals from clinical specimens is often overlooked (64). However, under an altered host immunologic state, a disrupted microbiome, or other factors that may predispose the host to microbial invasion, many common taxa within the skin microbiome demonstrate pathogenic behavior (65). The range of severity caused by opportunistic commensal control can vary significantly, from inflammation attributed to *Malassezia* and *Cutibacterium* to serious and occasionally lethal infection caused by *Corynebacterium* and *Staphylococcus.* Therefore, in the clinic, care should be taken to ensure that skin commensal isolates are not preemptively classified as contaminating skin flora.

Many species of the *Corynebacterium* genus are common members of the human skin microbiome (32), and therefore they are frequently classified as normal flora (64). However, Corynebacteria can cause serious disease, often nosocomial-associated or in immunocompromised individuals (66). In particular, Corynebacterium striatum has been implicated in a wide range of infections and is an emerging multidrug-resistant pathogen (67–69), yet it has also been identified in the healthy skin microbiota (70) (Fig. 3). Demonstrating its potential pathogenicity, an *in vivo* murine model of impaired wound healing revealed that C. striatum altered epidermal physiology consistent with delayed healing (71), suggesting that rather than existing as a bystander, this microbe may enhance pathology in wounds and other diseases.

The coagulase-negative staphylococcal species (CoNS), which excludes S. aureus, can also frequently cause nosocomial infections (72). S. epidermidis, which is often viewed as a model skin commensal for its beneficial interaction and cooperation with the host immune system, is also the most common species in CoNS infection (72, 73). S. epidermidis can form highly resistant and chronic biofilms, particularly on medical implants, and maintains a low inflammatory profile (74, 75), preventing immune clearance. The microorganism is also a leading cause of neonatal sepsis (76). Therefore, under certain conditions, this typically mutualistic skin commensal can evade immune clearance, leading to significant morbidity and mortality (Fig. 3).

While Cutibacterium acnes is a dominant bacterial skin colonizer (32), this species has been implicated in the pathogenesis of many diseases and infections (77). The most common skin disease associated with *C. acnes* is acne vulgaris, which affects the pilosebaceous follicle and results in inflammatory and noninflammatory clinical lesions (78). Numerous studies have provided insight into the host immune response to *C. acnes*, often characterized by inflammation (78, 79), but the mechanisms through which *C. acnes* induces these responses is less understood. Recent efforts have provided evidence that porphyrin production by *C. acnes* may be a key mediator of inflammation, as acne-associated *C. acnes* strains were shown to produce significantly higher levels of porphyrins than health-associated strains produced (55, 56). Curiously, vitamin B_12_ was found to regulate porphyrin production but only in acne-associated strains, supporting the importance of studying the skin microbiota and its contribution to health and disease in an ecological context (Fig. 3).

Similar to *C. acnes*, commensal species from the fungal genus *Malassezia* have also been implicated in various skin diseases, as well as in more serious infection (80, 81) (Fig. 3). Pityriasis versicolor, a superficial fungal infection of the skin, is the only dermatosis conclusively shown to be caused by *Malassezia* spp. (82). Generally, the fungal overgrowth seen in pityriasis versicolor causes little to no inflammation, which is suggested to be attributed to the ability of *Malassezia*-produced indolic compounds to downregulate components of the inflammatory cascade (80, 83, 84). Conversely, *Malassezia* spp. have shown to exacerbate certain inflammatory dermatoses, including atopic dermatitis and seborrheic dermatitis (80). This inflammation has been attributed to IL-17- and IL-6-driven responses (85, 86) and may be linked to secretion of extracellular vesicles (86, 87). However, the microbe-host interactions that dictate *Malassezia* spp. commensalism versus pathogenicity have not been fully elucidated.

To assess the relative proportions of pathobionts within the healthy skin microbiome, the average abundances of select pathobionts (such as those described above) across the human body were calculated from published relative abundance data (70) and are presented in Fig. 3. Reflecting prior studies showing that the skin microbiome composition varies by skin site characteristics (70, 88), pathobiont distribution also varies according to the skin microenvironment. Notably, there is a dominance and cooccurrence of *Malassezia* spp. and *C. acnes* in sebaceous sites, which are often the location for skin disease attributed to these pathobionts. Fungal pathogens such as *Candida* and *Trichosporon* are also found within the healthy skin microbiome, although at relatively low abundance, suggesting that the skin may be well equipped to limit fungal pathogen colonization. Overall, the presence of pathobionts in the skin microbiome underscores the essential role of the skin in maintaining homeostasis while also conferring protection against microbial overgrowth or pathobiont-induced inflammation.

## DEFENSIVE SYMBIOSIS

As the first line of defense to the environment, the skin functions to protect from invading pathogens, with the physical, chemical, and immune properties of the skin playing essential roles. For example, the physical structure of the outermost skin layer, the stratum corneum, consists of tightly packed, terminally differentiated keratinocytes that are protected by cornified envelopes. These cornified keratinocytes are surrounded by a lipid-rich extracellular matrix, forming a “brick-and-mortar”-like formidable barrier to protect the host from external factors (6). Furthermore, the acidic pH of the skin surface, largely from the hydrolysis of epidermal phospholipids into free fatty acids (FFA) (89), as well as the low water content of the stratum corneum (90), functions to inhibit pathogen colonization. Chemically, the human skin also deploys an arsenal of antimicrobial peptides (AMP) that are active against diverse pathogens, including Gram-positive and -negative bacteria, fungi, viruses, and parasites (91). The dominant AMPs produced on the skin include cathelicidins and β-defensins (Fig. 2), but many other protein families exist that employ diverse killing strategies. It is suggested that this diversity contributes to limiting the evolution of microbial resistance (9).

### Indirect commensal-mediated protection.

Even though the skin provides a seemingly inhospitable environment for microbial growth, the skin microbiota have evolved to survive on the skin. Rather than merely coexisting with the host, they contribute to antimicrobial defense through both indirect and direct mechanisms. Indirectly, the cutaneous microbiota acts to competitively exclude potential pathogens through occupying the skin niche and through utilizing the available limited nutrients (89). In addition, products of microbial metabolism may also indirectly bolster the antimicrobial capacity of the skin. For example, lipase activity in numerous skin commensals, including *C. acnes*, S. epidermidis, and *Malassezia* spp., allows for the hydrolysis of sebum lipids to FFAs (92–96), which contributes to acidification of the skin. Moreover, hydrolysis of skin surface triacylglycerols to FFAs by the nasal commensal *C. accolens* was found to inhibit the growth of the pathogen Streptococcus pneumoniae (97), demonstrating that in addition to reducing skin pH, commensal-derived FFAs may have direct antimicrobial activity (Fig. 2). Indeed, host FFAs have been well documented for their antimicrobial activity (98–100) and have even been shown to induce sebocyte expression of the AMP β-defensin hBD-2 (101).

Skin cells constitutively produce low levels of AMPs during homeostasis, forming an antimicrobial shield at the skin surface. However, increased expression of AMPs can be induced by skin barrier breach or microbial stimuli, typically through signaling mediated by pattern recognition receptors or proinflammatory cytokines (91). While the upregulation of AMP expression in response to skin pathogens has been well characterized, commensals have also been demonstrated to induce host AMP production (102). Notably, the host AMP response to commensal colonization is distinct from that of pathogen colonization and has been demonstrated to promote pathogen clearance (29, 50, 103, 104). Because of the ability of specific commensals to modulate host AMP production, it has been suggested that AMP production reflects the composition of the skin microbiome across skin sites and time and likewise may also directly affect community structure (102). Further insight is needed for a more comprehensive understanding of how skin microbial communities as a whole modulate host AMP production. Also, as the majority of the described studies have been performed with the commensal S. epidermidis, the effects of other dominant skin taxa, such as *Corynebacterium* spp., on AMP induction are worth pursuing as well.

### Direct commensal-mediated protection.

The resident skin microbiota also contribute directly to colonization resistance through the production of their own antimicrobial molecules (Fig. 2). Bacteriocins, which are ribosomally synthesized, heat-resistant, and highly potent molecules that often inhibit the growth of closely related bacterial species (105), have been identified to be produced by numerous CoNS species (19, 106–109) and are likely produced by the majority of skin commensals. S. epidermidis produces numerous bacteriocins and phenol-soluble modulins that selectively kill skin pathogens but are not active against S. epidermidis itself (110). The serine protease Esp also contributes to the antimicrobial arsenal of S. epidermidis and has been shown to effectively reduce S. aureus nasal carriage in humans (111). Similarly, the novel nonribosomal peptide lugdunin, synthesized by Staphylococcus lugdunensis and active against numerous skin pathogens, also reduced S. aureus nasal carriage (112).

A remarkable testament to the defensive symbiosis between the human skin and its microbiota is the cooperation and synergism between host and microbial AMPs. For example, lugdunin was found not only to increase expression of the host AMP LL-37 but also to synergize with other host AMPs, inhibiting S. aureus (113). Cooperation between host and microbial AMPs has also been demonstrated for S. epidermidis phenol-soluble modulins (110, 114) and lantibiotics from S. epidermidis and Staphylococcus hominis (19). Therefore, through the cooperation between innate host defense mechanisms and the resident microbiota, the skin is able to effectively ward off unwanted pathogens and maintain integrity of the skin barrier.

### Potential of the skin microbiota as a reservoir of antibiotics.

The described antimicrobial mechanisms employed by skin commensals exemplify the potential antibiotic reservoir within the human microbiota. The antibiotic mupirocin is a potent topical antibiotic used to treat skin infections caused by staphylococcal or streptococcal pathogens (65, 115). This compound was originally isolated from Pseudomonas fluorescens, a species that can sometimes be found within the human skin microbiome (116). Interestingly, several skin commensals, including *Micrococcus* spp. and *Corynebacteria* spp., are intrinsically resistant to mupirocin (117). From both an ecological and therapeutic standpoint, this is a desirable feature of mupirocin because it allows for the selective targeting of potential pathogens while preserving the native microbiota. As antibiotic discovery efforts to identify novel compound structures from soil microbes prove increasingly challenging, the human microbiota presents a relatively unexplored source of chemical diversity. Metagenomic efforts to mine the human microbiome for biosynthetic gene clusters (BGC) encoding potential antibiotics have revealed that the skin is enriched with BGC compared to other ecosystems like the gut (118). The products encoded by the vast majority of these BGC remain uncharacterized and may represent novel bioactive molecules. The commensal microorganisms that inhabit the human body must not only compete with each other for niche occupancy but also ward off invading pathogens, including pathogens for which there is a dire need for effective antibiotic treatment. Therefore, as described in this review, the microbiota have evolved numerous mechanisms for microbial competition, the majority of which are likely undiscovered.

## THE ROLE OF THE MICROBIOME IN TISSUE REPAIR AND WOUND INFECTION

Despite centuries of medical research dedicated to preventing and treating skin wound infection, the role of the skin microbiome in wound healing and infection continues to be an active and growing area of research. This coincides with a global increase in the prevalence of chronic, nonhealing wounds, the most common affecting the lower extremities such as diabetic foot ulcers and venous leg ulcers, followed by pressure ulcers and nonhealing surgical wounds. Normal cutaneous wound healing progresses through a series of highly coordinated events to restore skin integrity and barrier function. When this process becomes uncoordinated, excess inflammation, impaired angiogenesis, and increasing microbial bioburden occur (Fig. 1).

An aging population coupled to high rates of obesity and vascular disease only serves to compound the problem. Diabetes is estimated to affect 10.5% of the population in the United States, and this number rises to a staggering 26.8% for individuals over the age of 65. Up to 25% of diabetics will develop an ulcer in their lifetime, and alarmingly, the 5-year mortality rate for a person with a diabetic foot ulcer is over 50% (119, 120). Older individuals are also at an increased risk for developing a nonhealing wound or skin infection due to the aging process resulting in changes to the skin architecture, such as thinning of the epidermis, loss of elasticity, and decreased collagen production (Fig. 1) (121).

### Chronic wound microbiomes.

Nonhealing chronic wounds are characterized by the assembly of a distinct microbiome within the wound tissue, cardinal signs of infection often absent (122). Over the past decade, several studies using amplicon sequencing of phylogenetic marker genes have determined that chronic wound microbiomes are diverse communities containing both bacteria and fungi derived from intact skin and the environment (71, 123, 124). One of the most prevalent species found in diabetic wounds is S. aureus, which can alter expression of regulatory microRNAs resulting in delayed wound healing via repression of DNA repair and deregulation of the inflammatory response (125). However, commensal skin bacteria are also common, highly abundant, and phylogenetically diverse (71, 126–129). For these reasons, diagnostics and precision treatment remain challenging. Some advances have been made with computational modeling approaches. For example, several studies have shown that modeling temporal dynamics of the overall community structure is more predictive of healing trajectories, whereas communities with little structural change and turnover through time (i.e., more stability) are associated with longer and slower healing wounds (124, 130, 131). Although a single causative infectious agent is challenging to identify from these types of wounds, there is widespread use of topical antimicrobial products and administration of systemic antibiotics. Yet, studies have shown repeatedly that antibiotics do little to disrupt or shift wound microbial communities (71, 124, 132–134). We have found that genes encoding antibiotic resistance genes are widespread within chronic wound microbiomes, with some metagenomes harboring resistance genes to more than 10 different classes of antibiotics (71).

More recently, application of shotgun metagenomics to chronic wound microbiomes has permitted researchers to examine chronic wound microbiomes with greater granularity. For example, through orthologous approaches, including shotgun metagenomic analysis, comparative whole-genome sequencing, and an *in vivo* murine model of wound healing, we have identified specific strains of S. aureus harboring multiple types of enterotoxins and antibiotic resistance genes within their genomes, and these specific variants are exclusively associated with nonhealing diabetic wounds. On the other hand, strains classified as “generalist” were found in both healing and nonhealing wound microbiomes and tended to encode more genes related to immune evasion. This suggests that species- and strain-level classification may be important for designing therapeutic strategies and further supports the growing evidence that strain variation is a major driving factor in functional adaptation within the human microbiome and subsequent health outcomes (88, 135–137). Strain variation in S. aureus has been implicated in other dermatological disease outcomes such as psoriasis and atopic dermatitis (135, 138). In diabetic wounds, only the specialist S. aureus strains were positively correlated with microbial communities dominated by anaerobic bacteria, and mixed consortia of anaerobic bacteria have recently been identified as markers of poor clinical outcomes (71, 131, 139).

### Microbe-microbe interactions and wound healing.

The microbial communities of chronic wounds and adjacent intact skin are not always clearly demarcated (139, 140). Historically, commensals are largely ignored in acute wounds, but there is mounting evidence that for chronic wounds they may be playing a larger role in driving infection dynamics and outcomes. For example, C. striatum produces an unknown secreted factor that inhibits the *agr* regulatory system controlling virulence factors in the pathogen Staphylococcus aureus (61). Thus, on intact skin, C. striatum serves to prevent colonization and infection. On the other hand, C. striatum has been reported as a multidrug-resistant emerging wound pathogen (67, 141), and it has been shown to result in a hyperproliferative epidermal layer in diabetic mouse wounds (71). A consequence of competition with S. aureus in wound tissue might be additional local tissue damage. As discussed above, S. epidermidis is a skin commensal that is also a causative agent of wound infection; however, some strains prime the immune system to accelerate wound healing. Simply put, in a wound environment, the activity of commensals is important, but the ecological role of commensals is nuanced and should not be ignored. A major advance would be to identify the secreted factors produced by these organisms to understand how they shape microbial communities and mediate dialog with host cells.

The impact of interactions between fungal and bacterial species with wound-healing outcomes is another area of emerging interest. In a longitudinal study of 100 diabetic foot ulcer (DFU) patients, we found that up to 80% of wounds have fungi present and that they primarily represent two groups, allergenic filamentous fungi such as *Cladosporium* spp. and opportunistic pathogenic fungi such as *Candida* spp. We found that the allergenic fungi are associated with low levels of inflammation and euglycemia, while the abundance of mixed pathogenic fungi is significantly associated with tissue necrosis and longer healing times. Wounds with high levels of necrosis and fungi also had higher proportions of mixed anaerobic bacteria. We demonstrated that fungi and bacteria can form intimate physical connections and dense biofilms. Consequently, DFU pathology is linked to mixed microbial communities of fungi and bacteria (123).

Building upon this work, we have shown that priority effects, or the impact of an early colonizer on a late colonizer, can significantly alter the community structure in mixed fungal-bacterial biofilms and even lead to the suppression of S. aureus growth. Moreover, bacterial competition occurs for binding to fungal structures within biofilms. This suggests that even when fungal species such as Candida albicans make up a small proportion of the overall community, they can stabilize the community by providing alternate binding sites for bacterial attachment and biofilm growth, leading to interspecies competition. We also discovered that the facultative anaerobe Citrobacter freundii triggers the yeast-hyphae switch in C. albicans and that cocultures of these two species result in enhanced neutrophil killing, a hallmark of excess inflammation in DFUs (142). Taken together, interspecies interactions within wound microbiomes are highly complex and driven by mechanisms we have yet to define.

## PERSPECTIVE

The skin is a remarkable and resilient organ that protects us from the outside world. It also provides diverse microbial habitats exhibiting distinctive microenvironments that serve to shape the composition of populating communities. While important advances have been made highlighting the role the skin microbiome plays in developing skin immunity and resistance to pathogens, there is much left to explore. The majority of studies have focused on dominant skin species in the genera *Staphylococcus* and *Cutibacterium*, two groups containing both beneficial and pathogenic species. But what about other major genera found on the skin? Human skin is dominated by the phylum *Actinobacteria*, with species spanning but not limited to the *Corynebacterium*, *Kocuria*, *Micrococcus*, and *Brevibacterium* genera. All genera have reported examples of infection, but for the most part they are understudied and their roles on the skin and within the microbiome are largely unknown. Even well-studied species, such as S. epidermidis, leave many unanswered questions regarding the molecular mechanisms driving interactions and immunological responses described above. The skin virome is another important but poorly understood component of the microbiome, even though it may contribute to shaping skin microbial communities and influencing skin health.

Looking forward, research aimed at identifying the signals and response pathways that maintain the delicate balance between commensalism and pathogenicity will undoubtedly lead to novel therapeutics and pave the way for microbiome engineering on the skin surface. A hurdle to understanding these signals and responses is the prediction of absolute microbial abundance and physiological concentrations of metabolites within the microenvironment, which is a current limitation of using relative abundance data derived from sequencing. Excitingly, the resurgence of “culturomics” and advances in proteomics and mass spectrometry can overcome these limitations to allow for the study of molecules produced and shared within skin microbial communities (143, 144). These tools facilitate the development of skin models mimicking nutrient availability on the skin to probe how the chemical environment of different skin sites and conditions influences microbial growth (absolute abundance), metabolism, and host responses. As exemplified in this review, the identification of defined metabolites and species that mediate microbe-microbe and microbe-host interactions is essential for understanding the relationship between the skin and its microbiome at a fundamental level. Application of this knowledge may lead to engineered microbial communities on the skin using a probiotic-based strategy, targeting diverse dermatological diseases from atopic dermatitis to wound healing. In the future, an emphasis should be placed on studying these interactions with increasing complexity to accurately reflect the complexity and diversity of the human skin microbiome.
